# Imaging for Metastasis in Prostate Cancer: A Review of the Literature

**DOI:** 10.3389/fonc.2020.00055

**Published:** 2020-01-31

**Authors:** Anthony Turpin, Edwina Girard, Clio Baillet, David Pasquier, Jonathan Olivier, Arnauld Villers, Philippe Puech, Nicolas Penel

**Affiliations:** ^1^Department of Medical Oncology, CHU Lille, Lille, France; ^2^Univ. Lille, CNRS, Inserm, CHU Lille, Institut Pasteur de Lille, UMR9020 – UMR-S 1277 – Canther - Cancer Heterogeneity, Plasticity and Resistance to Therapies, Lille, France; ^3^Medical Oncology Department, Centre Oscar Lambret, Lille, France; ^4^Nuclear Medicine Department, CHU Lille, Lille, France; ^5^Academic Department of Radiation Oncology, Centre Oscar Lambret, Lille, France; ^6^CRISTAL UMR CNRS 9189, Lille University, Villeneuve-d'Ascq, France; ^7^Department of Urology, CHU Lille, Lille, France; ^8^Department of Radiology, CHU Lille, Lille, France

**Keywords:** prostate cancer, choline-PET, fluciclovine-PET, PSMA-PET, bone scan, MRI, staging

## Abstract

**Background:** Initial staging and assessment of treatment activity in metastatic prostate cancer (PCa) patients is controversial. Indications for the various available imaging modalities are not well-established due to rapid advancements in imaging and treatment.

**Methods:** We conducted a critical literature review of the main imaging abnormalities that suggest a diagnosis of metastasis in localized and locally advanced PCa or in cases of biological relapse. We also assessed the role of the various imaging modalities available in routine clinical practice for the detection of metastases and response to treatment in metastatic PCa patients.

**Results:** In published clinical trials, the most commonly used imaging modalities for the detection and evaluation of therapeutic response are bone scan, abdominopelvic computed tomography (CT), and pelvic and bone magnetic resonance imaging (MRI). For the detection and follow-up of metastases during treatment, modern imaging techniques i.e., choline-positron emission tomography (PET), fluciclovine-PET, or Prostate-specific membrane antigen (PSMA)-PET provide better sensitivity and specificity. This is particularly the case of fluciclovine-PET and PSMA-PET in cases of biochemical recurrence with low values of prostate specific antigen.

**Conclusions:** In routine clinical practice, conventional imaging still have a role, and communication between imagers and clinicians should be encouraged. Present and future clinical trials should use modern imaging methods to clarify their usage.

## Introduction

Prostate cancer (PCa) is the first cancer in terms of incidence in men (1). Most PCa are curable but metastatic forms are associated with lower survival (2), hence the need for imaging to detect and to follow metastases evolution during treatment.

The use of various different imaging modalities is an important source of heterogeneity in the diagnosis and treatment of PCa patients. This variability is largely due to the rapid evolution of knowledge and the availability of new drugs and high-technology imaging techniques, such as prostatic magnetic resonance imaging (MRI) and the emergence of new radiotracers. This issue was highlighted in the latest international recommendations from the expert consensus panel of the Advanced Prostate Cancer Consensus Conference (3, 4), which noted that an updated consensus is required due to the low level of evidence from clinical studies in radiology and nuclear medicine, which were mainly retrospective.

Recently, a multidisciplinary panel of international experts convened at the European Association of Nuclear Medicine (EANM) Focus 1 meeting produced a comprehensive series of statements on prostate cancer imaging and therapy with radiopharmaceuticals (5). Notably, bone scintigraphy and CT have never been recommended for the majority of patients by experts despite the fact that these methods are still largely included in most clinical guidelines. In another recent consensus statement, provided by the European Organization for Research and Treatment of Cancer (EORTC) imaging group following a review of the role of modern imaging for optimal identification of oligometastatic disease (6), an imaging trial design was proposed.

In light of the latest consensus recommendations, we aimed to assess the rationale for the main imaging methods available in routine clinical practice for the diagnosis of metastatic PCa, including initial staging and therapeutic response, in order to enhance the clinical care of PCa patients.

## Methods

### Study Design

This review of the literature, aimed at analyzing all studies that investigated the use of imaging as a basis for the staging of metastatic PCa or the assessment of patients treated for metastatic PCa.

### Literature Search

The studies were screened manually by two independent authors (AT and EG) on the basis of the study title and abstract. The relevant studies were abstracted according to the eligibility criteria. The data of interest were independently extracted from the selected studies by two authors (AT and EG). All the study details (year of publication, study primary author, and trial design) were extracted. Extracted data were double-checked by a third reviewer (NP). Discrepancies were resolved by discussion with all the authors.

The keywords “metastatic prostate cancer” were used in the systematic search conducted between October 1, 2018 and December 31, 2019, and associated with “first line” [183], “staging” [354], “MRI” [76], “PET” [184], “bone scan” [117], and “imaging” [568]. For the localized and locally advanced PCa sections, we also searched for “prostate cancer” and “seminal vesicles” and “MRI” [127]; “prostate cancer” and “seminal vesicles” and “PET” [17]; “prostate cancer” and “prostate bed” and “MRI” [33]; and “prostate cancer” and “prostate bed” and “PET” [66]. The methodological search was performed through the online database, MEDLINE. Additionally, the references in each eligible article were examined. Furthermore, relevant manuscripts were screened when a positive match was identified.

### Inclusion and Exclusion Criteria

Only original manuscripts and reviews published in indexed and peer-reviewed journals and written in English between August 1999 and December 2019 were considered. Cross-sectional studies, case reports, published abstracts, dissertation materials, and conference presentations were excluded. Of 1,725 potential articles from the literature search, 105 were selected, including 16 literature reviews, 5 meta-analyses, 11 guidelines or position papers, and 73 original articles.

## Results

From the current international guidelines, we searched the literature for the levels of evidence concerning the use of different imaging modalities in routine clinical practice.

### Search for Metastases in Localized and Locally Advanced Prostate Cancers

The search for metastases was performed based on the presence of clinical symptoms, from the diagnosis of localized PCa in asymptomatic patients, or in the follow-up of treated PCa. Lymph node (LN) or bone metastases are rarely detected in this context (7).

In patients followed-up for localized PCa, metastases should be sought according to the risk group defined by the National Comprehensive Cancer Network (NCCN) or by the European Association of Urology (EAU) guidelines (6), to estimate the 5-year biological relapse risk. These guidelines are heterogeneous across the world. Hence, it is of interest to provide answers for routine clinical practice to the following question: How can metastases in LN, bone, or locoregional disease in seminal vesicles (SVs) be better diagnosed?

#### Is There Disease in the LN (Table 1)?

Computed tomography (CT) scans are mainly performed to diagnose LN involvement. Nevertheless, there is difficulty with CT scan validation, either at initial diagnosis or for recurrence.

**Table 1:** Sensitivity and specificity of currently available functional and targeted imaging methods for LN staging of PCa.

**Type of imaging**	**Reference and type**	**Number of patients**	**Se (%)**	**Sp (%)**
CT-scan	Abuzallouf et al. (8) (Review)	*N* = 4,264	7	100
	Hövels et al. (9) (Meta-analysis)	18 studies, *N* = 1,024	42	82
Choline-PET/CT	Mapelli and Picchio (10) (Meta-analysis)	5 studies, *N* = 177	94–100	66–99.7
PSMA-PET/CT	Maurer et al. (11) (Original article)	*N* = 130	65.9	98.9
	Kim et al. (12) (Meta-analysis)	6 studies, *N* = 298	71	95
MRI	Mapelli and Picchio (10) (Meta-analysis)	5 studies, *N* = 177	18.8–69.7	78.6–97.6
	Hövels et al. (9) (Meta-analysis)	10 studies, *N* = 628	39	82
MRI with magnetic nanoparticles	Harisinghani et al. (13) (Original article)	*N* = 33	90.5	97.8

*Choline-PET/CT, choline positron emission tomography/computed tomography scan; CT-scan, computed tomography scan; PSMA-PET/CT, prostate-specific membrane antigen-positron emission tomography/computed tomography scan; MRI, magnetic resonance imaging; Se, sensitivity; Sp, specificity*.

In a single >10-year-old review manuscript, the overall sensitivity, specificity, positive predictive value (PPV), and negative predictive value (NPV) of LN metastases detection with CT were 16, 100, 85, and 100%, respectively (8). Among 25 studies, LN involvement, detected by CT, was documented to affect ~0 and 1.1% of patients with prostate specific antigen (PSA) <20 and ≥20 ng/mL, respectively. The CT detection rate was 0.7 and 19.6% in patients with localized and locally advanced diseases, respectively. Detection rates in patients with Gleason scores ≤ 7 and ≥8 were 1.2 and 12.5%, respectively (8).

Some encouraging results support the role of choline positron emission tomography (PET)/CT in LN staging for selected patients with high risk of LN invasion. Choline-PET/CT is considered a reliable tool for LN staging of PCa because it has good sensitivity (94–100%) and specificity (66.7–99.7%), although some studies reported a lower specificity than for MRI (78.6–97.6%), and no consensus exists regarding whether choline-PET/CT or MRI should be used for LN staging (10).

In a large-scale meta-analysis, the pooled sensitivity and specificity of conventional MRI for detecting pelvic LN metastases from PCa were ~39 and 82%, respectively (9). In preoperative patients, there was improved detection utilizing diffusion weighted imaging (DWI) when compared to conventional cross-sectional imaging techniques, with the detection of LN metastasis in 64–79% of cases (14). The current threshold for reporting involved nodes on multiparametric MRI are those measuring ≥8 mm on the short axis (15). However, metastatic LNs are not always enlarged, and standard pelvic MRI cannot detect micrometastases. Lymphotropic nanoparticles have demonstrated superior sensitivity than conventional MRI (90.5 vs. 35.4%) in the detection of LN metastases in PCa (13). Magnetic resonance lymphography utilizing ultra-small super paramagnetic iron oxide (USPIO) has shown high sensitivity and specificity in the detection of normal-sized LN containing metastatic disease. While USPIO is efficacious, it is not widely available (13, 16).

Fluorodeoxyglucose 18 (18FDG) PET has shown promise in clinical practice, with increased sensitivity in undifferentiated forms. A United States (US) registry study showed a change in therapeutic management for 32% of patients with systemic FDG-PET scans in the initial staging (17). In a small study, among patients with a high-grade PCa at biopsy, FDG-PET/CT could improve pre-treatment prognostic stratification by predicting primary PCa pathological grade and survival probability following radical prostatectomy (RP) (18).

Prostate-specific membrane antigen (PSMA) is overexpressed in PCa cells, allowing for detection using PET/CT imaging with gallium 68-labeled PSMA ligands (PSMA-PET/CT). This is a high-sensitive test for metastasis detection, but it is recent and still being evaluated. In a retrospective analysis of 130 consecutive patients with primary, intermediate-risk to high-risk PCa who underwent RP with template pelvic LN dissection, there was a sensitivity of 65.9% and specificity of 98.9% for LN staging with PSMA-PET/CT. The specificity of CT was significantly lower the PSMA-PET (11). Recently, in a six-study meta-analysis, the overall sensitivity and specificity for PSMA-PET/CT was 71 and 95%, respectively (12). Hence, PSMA-PET is the most encouraging imaging modality for the detection of disease in LNs.

#### Is There Disease in Seminal Vesicles (Table 2)?

The combination of a tumor at the prostate base extending beyond the capsule, and low signal intensity within SVs in a background of high signal fluid on T2-weighted images is highly predictive of SV invasion (SVI) using multiparametric MRI (22). There is good concordance between MRI and histopathology in surgical patients, but this requires that the radiology team be trained (22, 23). Indeed, in 79 Brazilian patients who underwent multiparametric MRI, only 5% had an SVI and 4% had LN invasion. With surgical specimens, SVI, with a sensitivity of only 19.4% and a specificity of 100%, was found in 26.6% of specimens (23).

**Table 2:** Sensitivity and specificity of currently available functional and targeted imaging methods for detection of SVI in PCa.

**References**	**Type of imaging**	**Number of patients**	**Se (%)**	**Sp (%)**
Grivas et al. (19)	MRI	527	75.9	94.7
Pinaquy et al. (20)	Choline-PET/CT	47	36	98
Fendler et al. (21)	PSMA-PET/CT	21	73	100

*Choline-PET/CT, choline positron emission tomography/computed tomography scan; PSMA-PET/CT, prostate-specific membrane antigen-positron emission tomography/computed tomography scan; MRI, magnetic resonance imaging*.

In a more robust study, by rereading the clinico-radiological data of 527 patients who had a robot-assisted RP, and comparing the SVI imaging reports with histological analysis, 54 (10%) patients were identified as having SVI. Overall, the sensitivity, specificity, PPV, and NPV for SVI detection with MRI were 75.9, 94.7, 62, and 97%, respectively. Based on a sub-analysis, radiologists with greater expertise demonstrated improved accuracy, with a sensitivity, specificity, PPV, and NPV of 85.4, 95.6, 70.0, and 98.2%, respectively. In a multivariate analysis, MRI provided added diagnostic value to PSA, above that of the clinical/Partin-based SVI-prediction models alone (19).

Regarding the contribution of choline-PET/CT for tumor staging, MRI with DWI showed a better performance with improved specificity for sextants analysis (69 vs. 44%) and a better sensitivity to detect SVI (73 vs. 36%) in 47 patients who underwent choline-PET/CT and MRI followed by surgical treatment (20). In another study, PSMA-PET correctly detected SVI with 71% accuracy in a 21-patient study with biopsy-proven PCa (21). Therefore, SVI is best detected by T2-weighted (T2W) sequence MRI (24).

#### Is There Disease in Bones (Table 3)?

**Figure 1:**
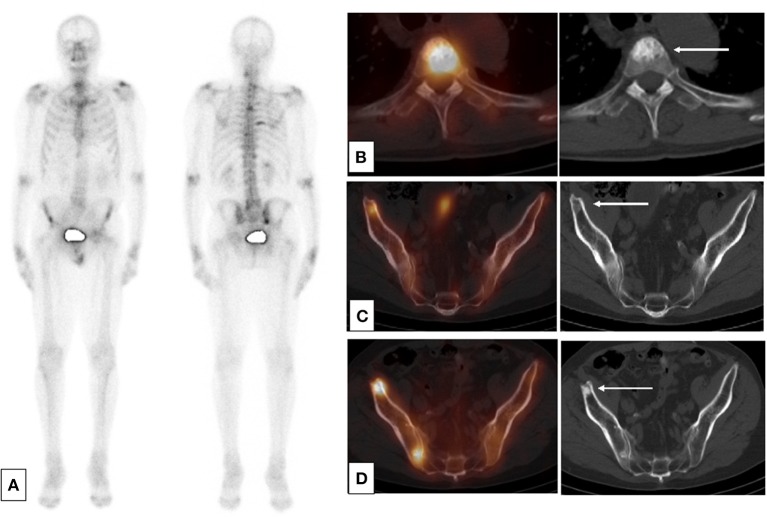
SPECT-CT: condensation of bone lesions under treatment. Occurrence of a prostate adenocarcinoma, Gleason 6 (3 + 3) on a biopsy, unoperated, treated by hormone therapy and HIFU therapy in a patient. Progressive re-elevation of the PSA 2 years after the cessation of hormone therapy. **(A)** Baseline planar bone scan **(B,C)** Baseline SPECT-CT and CT (axial slices) of lesions of T4 **(B)** and right ilium **(C)**. **(D)** SPECT-CT and CT after 1 year of treatment by leuprorelin acetate showing an osteosclerotic reaction in the right ilium.

Disease progression on whole body bone scintigraphy using 99mTc-labeled diphosphonate remains the main criterion recognized by the Food and Drug Administration (FDA) in the US for the evaluation of bone response. A 12-article meta-analysis of bone scan (BS) measured on a per-patient basis found a pooled sensitivity of 0.79 (95% CI: 0.73–0.83) and a pooled specificity for bone metastasis detection of 0.82 (95% CI: 0.78–0.85). On a per-lesion basis, the pooled sensitivity and specificity for BS were 0.59 (95% CI: 0.55–0.63) and 0.75 (95% CI: 0.71–0.79), respectively (25). However, sensitivity and specificity were improved when coupled with low-dose CT (26). The performance was also improved by using single-photon emission computed tomography (SPECT) with CT (SPECT-CT) (Figure 1). Thus, the sensitivity of 70% in BS increased to 87–92% with SPECT-CT (26). BS does not directly visualize the metastasis but rather visualizes the osteoblastic reaction to the presence of tumor cells. It detects <1% of bone metastases in patients with PSA <20 ng/mL (27, 29). Metastases were detected in 2.3% of patients having a PSA <10 ng/mL, 5.3% of patients having a PSA level between 10.1 and 19.9 ng/mL, and 16.2% of patients with a PSA between 20.0 and 49.9 ng/mL (8). The sensitivity and specificity of bone CT for the detection of bone metastases is 56 and 74%, respectively (30), when bone CT is indicated.

**Table 3:** Sensitivity and specificity of currently available functional and targeted imaging methods for bone staging of PCa.

**Type of imaging**	**Reference and type**	**Number of patients**	**Se (%)**	**Sp (%)**
Bone scan	Shen et al. (25) (meta-analysis)	18 studies, *N* > 2,291	79	82
Choline-PET/CT			87	97
MRI			95	96
SPECT-CT	Behesti et al. (26) (Review)	Review	87–92	91
Bone CT	O'Sullivan et al. (27) (Review)	Review	56	74
NaF-PET/CT	Behesti et al. (28) (Review)	Review	100	100

*Bone CT, bone computed tomography scan; Choline-PET/CT, choline positron emission tomography/computed tomography scan; CT-scan, computed tomography scan; MRI, magnetic resonance imaging; NaF-PET/CT, 18F-sodium fluoride positron emission tomography/computed tomography scan; PSMA-PET/CT, prostate-specific membrane antigen-positron emission tomography/computed; Se, sensitivity; Sp, specificity; SPECT-CT, single photon emission computed tomography*.

**Figure 2:**
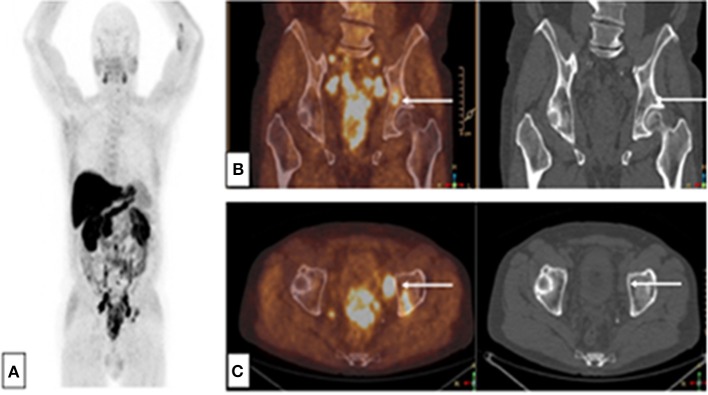
Choline-PET-CT: Single bone lesion of the left acetabulum, without CT abnormality. Initial assessment of a patient with immediately metastatic prostate adenocarcinoma with bone and node lesions. Gleason 8 (4 + 4), cT3, PSA = 36 ng/mL, **(A)** MIP reconstruction, **(B)** PET-CT and CT frontal slices, **(C)** PET-CT and CT axial slices.

PET/CT using 18F- or 11C-labeled choline (choline-PET/CT) dramatically improved the detection of infra-radiological bone metastases, with a sensitivity >90–95% and a specificity ranging from 92 to 99% (31, 32) (Figure 2). Indeed, choline is a precursor of phospholipids constituting the cellular membrane, and radio-labeled choline incorporation is increased in cell proliferation. Therefore, choline-PET/CT can distinguish between malignant and degenerative bone defects, which are not choline avid, even though choline can accumulate in recent traumatic bone lesions.

In a meta-analysis of 27 studies involving advanced PCa patients, Shen et al. showed that MRI was superior to choline-PET and bone scintigraphy for detecting bone metastases, with a sensitivity of 97% for MRI vs. 91% for choline-PET/CT vs. 79% for bone scintigraphy in and a specificity of 95% for MRI vs. 99% for choline-PET/CT vs. 82% for bone scintigraphy (25). When a doubt persists about bone slices, suggesting traumatic lesions or a flare reaction with an increase in osteoblastic activity, the results must be confirmed by bone MRI or bone CT.

Nevertheless, in modern and more robust prospective studies, choline-PET (33) and PSMA-PET (34) were significantly better especially when conventional imaging such as MRI were negative (35) or non-contributive (36).

Sodium fluoride (NaF) has high affinity for osteoblasts and reflects the same phenomenon as bone scans. NaF-PET/CT can be used in the evaluation of primary and secondary bone malignancies, highlighting increased bone remodeling and allowing assessment of response in PCa (28). Several studies suggest that NaF-PET is superior to bone scan for the detection of bone metastases if PET is associated with CT (100% sensitivity and 100% specificity vs. 70 and 57%, respectively, for bone scintigraphy alone) (37).

Once again, bone MRI is a useful imaging technique for detecting bone metastases. Using a whole-body-MRI (WB-MRI), any pairwise combination of T1-weighted, short-TI Inversion Recovery (STIR), and DWI, has high diagnostic accuracy (38). The main limitations for using this exam in routine practice are the availability of WB-MRI and the high cost of the equipment (16).

Prostate physicians should discuss the association between MRI and bone CT in multidisciplinary consultations to better diagnose and treat bone metastasis. Choline-PET and PSMA-PET are valuable tools particularly when conventional imaging is negative.

### Search for Metastases in Recurrent Disease: Restaging

About 30% of patients treated radically for high or very high risk PCa have biochemical recurrence (BCR) (39), and with modern imaging, the diagnosis of oligometastatic PCa has become more common (6). Current guidelines from NCCN and EAU (6) but stay heterogeneous across different parts of the world. For example, National Comprehensive Cancer Network guidelines consider 18F fluciclovine-PET-CT for prostate cancer biochemical recurrence after radical prostatectomy^1^ (NCCN guidelines), whereas European Association of Urology guidelines recommend prostate-specific membrane antigen PSMA-PET/CT (39). Tables 4, 5 summarize the literature on the following issues.

**Table 4:** Currently available functional and targeted imaging methods for detection of suspected recurrence in PCa with biological relapse in patient-based analyses.

**References**	**Type of imaging**	**Number of patients**	**Diagnosed recurrence (%)**	**LR (%)**	**BR (%)**	**LNR (%)**
Gauvin et al. (40)	Choline-PET	*N* = 60	63.3	26.6	8	20
Schwenck et al. (41)	Choline-PET	*N* = 123	79	–	64	71
	PSMA-PET		83		98	94
Uprimny et al. (42)	PSMA-PET	*N* = 203	62.1	12.8	17.2	39.9
Afshar-Oromieh et al. (43)	PSMA-PET	*N* = 1,007	79.5	9.6	13	26.1
Calais et al. (44)	PSMA-PET	*N* = 50	56	14	8	30
	Fluciclovine-PET		26	18	0	8
Mena et al. (45)	DCFBC-PET	*N* = 68	60.3	44	16.6	57
Robertson et al. (46)	WB-MRI	*N* = 76	21	9	9	8

*Choline-PET, choline C-11 positron emission tomography; DCFBC-PET, DCFBC positron emission tomography; PSMA-PET, prostate-specific membrane antigen-positron emission tomography; WB-MRI, whole body magnetic resonance imaging; LR, local recurrence; BR, bone recurrence; LNR, lymph node recurrence*.

**Table 5:** Sensitivity and specificity of currently available functional and targeted imaging methods for detection of suspected recurrence in PCa with biological relapse in patient-based analyses.

**References**	**Type of imaging**	**Number of patients**	**Se (%)**			**Sp (%)**		
			**PB**	**LN**	**BM**	**PB**	**LN**	**BM**
Huysse et al. (36)	Bone MRI	*N* = 64	–	–	100	–	–	96
	Choline-PET				87			100
Kitajima et al. (47)	MRI	*N* = 115	88.5	64	87.5	84.6	85	96.2
	Choline-PET		54.1	90	81.3	92.3	100	98.7
Afshar-Oromieh et al. (43)	PSMA-PET	*N* = 1,007	79.5%	–				
Zacho et al. (48)	MRI	*N* = 60	–	–	25	–	–	87
	PSMA-PET				80			100
	NaF-PET				90			98
Bach-Gansmo et al. (49)	Fluciclovine-PET	*N* = 596	88.1	–	–	32.6	–	–

*PB, prostate bed; LN, lymph node; BM, bone metastasis; Bone MRI, bone magnetic resonance imaging; Choline-PET, choline C-11 positron emission tomography; MRI, magnetic resonance imaging; NaF-PET, 18F-sodium fluoride positron emission tomography; PSMA-PET, prostate-specific membrane antigen-positron emission tomography; Se, sensitivity; Sp, specificity*.

#### Is There Residual Tumor in the Prostate Bed?

If abnormalities were best appreciated on T2W axial images as focal hypointense lesions (50), dynamic contrast-enhanced imaging (DCE-MRI) or DWI in combination with T2W at 3T with a phased-array coil, appears to be more useful than T2W alone in evaluating suspected soft tissue lesions of the prostate bed after RP (51).

In a study of 76 consecutive patients with suspected PCa recurrence after RP, WB and dedicated prostate MRI was completed successfully in all patients. Suspected disease recurrence was identified in 21% (16/76) of patients, including local recurrence in the prostate bed in 8% (46).

Choline-PET/CT and MRI showed comparable results in terms of sensitivity, specificity, PPV, and NPV for PCa characterization when restaging PCa patients with BCR following RP (52). The sensitivity and specificity of choline-PET were 73.3 and 75.6%, respectively (53). In a study by Kitajima et al. the sensitivity and specificity for loco-regional recurrence detection were 54.1 and 92.3% for choline-PET/CT, and 88.5 and 84.6% for MRI, respectively (47). Choline in the prostate bed and bladder-urethral junction along the midline must be considered suspicious for local relapse in patients treated radically for PCa, especially if they are presenting with a PSA level >1 ng/mL (53).

PET/MRI suffers from limited availability, and technical modifications in PET/CT protocols may improve localization of prostate bed disease. In the case of PSMA-PET/CT, early dynamic imaging led to an increase in the detection rate from 20.3 to 29.7% in a subgroup of 64 patients with BCR (42). Similarly, early pelvic imaging was found to be of benefit in another study of 203 patients with BCR. The number of equivocal findings on PET scans 60 min post injection was significantly reduced with the help of early imaging (15.8 vs. 4.5%; *P* < 0.001) (42). DCE imaging performed the best for suspected prostate bed recurrence, detecting correlates for 87.5% (14/16) of PSMA-positive prostate bed foci (54). With 18F-DCFBC PET/CT, using another PSMA-targeted PET agent, recurrences were detected in 60.3% of patients with BCR, but the results were dependent on PSA levels. Above a threshold PSA value of 0.78 ng/mL, among a total of 79 18F-DCFBC avid lesions, 30 were in the prostate bed (45). Moreover, as salvage radiotherapy is most effective at low serum PSA values, PSMA-PET/CT imaging could be used to optimize radiotherapy planning by defining the target lesions or areas that are most appropriate for boost radiotherapy (55).

As fluciclovine is an 18F radiotracer, an on-site cyclotron is not required for its production, which is useful in clinical routine. In a prospective trial of 100 patients, there was a statistically significant difference in terms of detection of local relapse between 11C-choline and 18F-fluciclovine (*p* < 0.0001) (56). Nevertheless, in patients with an intact prostate, fluciclovine-PET demonstrates high sensitivity and low specificity in identifying local recurrent disease with a sensitivity of 88–90% and specificity of 32–40% (57). In a new a single-center study including 50 participants, Calais et al. tested PSMA-PET and fluciclovine-PET head-to-head in the same patients and compared their cancer detection rates in patients with low serum PSA levels (<2 ng/ml) after radical prostatectomy. Detection rates for prostate bed recurrence did not differ significantly between the two radiotracers (18 vs. 14%; *p* = 0.73) (44).

#### Is There LN Involvement?

In the case of biological recurrence, 11C-choline PET/CT is superior for detecting pelvic LN metastases when restaging prostatectomy patients with suspected recurrent disease (47). Choline-PET/CT showed good sensitivity and specificity for the early detection of LN metastases (58, 59), especially in cases of failure of conventional imaging to detect metastases.

In the study by Kitajima et al., the sensitivity and specificity for LN recurrence detection were 90% and 100% for choline-PET/CT, and 64% and 85% for MRI, respectively (47). Choline-PET/CT performances are better for higher PSA levels and higher PSA doubling time. Various PSA cut-offs, between 1.4 and 2.6 ng/mL, have been suggested to determine the optimal timing to perform choline-PET/CT (40). Previous studies have indicated that recurrence can be identified with a detection rate of 19% (60), 26% (61), or 31% (62) in patients with PSA <1 ng/mL. This threshold can be lowered when the PSA velocity is >1 ng/mL/year, if PSA doubling time is <6 months, or if androgen deprivation therapy is ongoing (62–64). In a study of 123 patients with relapsed PCa, PSMA-PET/CT showed a higher LN detection rate than choline-PET/CT (94 vs. 71%, *p* < 0.001), even though ultimately there were mismatches for both tracers (41).

In 2016, the U.S. Food and Drug Administration found the accuracy of fluciclovine-PET to be superior to that of other molecular imaging techniques, including Choline-PET and subsequently granted approval for its use in PET of recurrent prostate cancer.

A large multisite study of 596 patients found a high PPV of 92.3% in the detection of extraprostatic disease (49). In patients with PSA values <1 ng/mL, fluciclovine has relatively low sensitivity for extraprostatic disease ranging from 21 to 39%.

The results of the head to head Calais's trial, revealed that cancer detection rates of BCR per patient were significantly lower with fluciclovine PET than with PSMA-PET (26 vs. 56%; *p* = 0.0026). On further analysis, fluciclovine detection rates of BCR were significantly lower than PSMA-PET detection rates for the pelvic lymph node region (8 vs. 30%; *p* = 0.0034) and for any extrapelvic lesions (0 vs. 16%; *p* = 0.0078) (44).

#### Is There Bone Disease?

When PSA is high (>10 ng/mL), standard imaging i.e., CT scan and bone scan is usually sufficient to confirm the metastatic status (63). However, in cases of BCR and a low PSA level, PET/CT may be indicated to view the recurrence site and search for distant metastases with more accuracy than conventional imaging (39, 65).

In the study by Kitajima et al., the sensitivity and specificity for bone recurrence detection were 81.3 and 98.7% for choline-PET/CT, and 87.5 and 96.2% for MRI, respectively (47). In another study of 64 patients with biological relapse, the sensitivity of MRI was significantly better compared to that of choline-PET/CT (*p* = 0.031), and the specificity did not differ significantly (*p* = 0.125) (36).

In a single-center retrospective study, of 106 patients with metastatic prostate cancer who had both fluciclovine-PET/CT and bone scan within 3-month interval, the sensitivity, specificity, positive predictive value and negative predictive value for bone scan were 79, 86, 45, and 96%, respectively; and 100, 98, 89, and 100% in fluciclovine PET/CT, respectively. These results demonstrated that fluciclovine-PET/CT detected more bone metastases than bone scan. Importantly, there were no lesions identified by bone scan that was missed by fluciclovine-PET/CT (66).

Currently, recurrent PCa is the main indication for the use of PSMA-PET, and the majority of published data focus on this setting. In patients who have undergone RP, PSMA-PET/CT improves detection of metastatic PCa compared with conventional cross-sectional imaging or bone scintigraphy. Furthermore, it increases the detection of lesions even at serum PSA values <0.5 ng/mL compared with conventional imaging or PET examination with different tracers. In a study including over 1,000 patients, PSMA-PET/CT detected at least one recurrence site in 801 patients (79.5%), with high detection rates even for low PSA levels, at 46 and 73% for PSA <0.5 ng/mL and PSA between 0.5 and 1 ng/mL, respectively (43). In another study of 123 patients with relapsed PCa, PSMA-PET showed a higher detection rate than choline-PET/CT for bone lesions (98 vs. 64%), even though ultimately there were mismatches for both tracers (41). PSMA-PET/CT and 18FNa-PET/CT methods were comparable and performed significantly better than DW600-MRI, which was less adequate for diagnosing bone metastases when conducted in accordance with the European Society of Urogenital Radiology guidelines (48).

In the Calais's trial, no significant differences were observed between different levels of extrapelvic disease (M1a, M1b, and M1c) between fluciclovine-PET and PSMA-PET, most probably because the sample sizes were too small (*N* = 50) (44).

### Monitoring of Metastatic PCa

#### Extent of Disease

New imaging modalities such as MRI, choline, fluciclovine, and PSMA-PET/CT, appear to have excellent sensitivity and specificity for lesion detection, although they have not yet been adequately tested in formal clinical trials. For example, the lymph node detection sensitivity of choline-PET/CT varies from 41.5 to 56%, while the specificity varies from 94 to 98.8%, with higher sensitivity observed for the detection of LN >5 mm, and particularly outside lymphadenectomy territories (67–69). Data on the impact of this gain in sensitivity on patient care is lacking. Moreover, the CT scan of the past bears little resemblance of the CT scanning we use today, particularly with resolution. And PSMA-scans vary in the radio-isotope used as well as the molecules used as imaging probes. These PSMA-scans are generally undertaken in conjunction with an axial imaging modality.

Currently, the therapeutic decision is based on the experience of clinicians, the status of the disease, and patient comorbidities (63). The St Gallen consensus proposes thoracic and abdominopelvic CT as well as a bone scintigraphy before the beginning of a new treatment and for use during patient follow-up (3, 4). In previous clinical trials among metastatic PCa patients, heterogeneity in terms of imaging is the rule. The various modalities employed at initial staging and evaluation of treatment response are summarized in Table 6 for hormone-sensitive PCa, Table 7 for first-line castration-resistant PCa, and Table 8 for castration-resistant PCa in subsequent lines.

**Table 6:** Assessment exams in former phase III trials.

	**GETUG-15** **(****70****)**		**CHAARTED** **(****71****)**		**LATITUDE** **(****72****)**	
	**Type of evaluation**	**Time**	**Type of evaluation**	**Time**	**Type of evaluation**	**Time**
Clinical exam	×	Baseline then/3 weeks in the docetaxel group/3 months in the ADT group	×	Baseline then/3 weeks in the docetaxel group/3 months in the ADT group	–	–
Bone scan	–	–	×	Baseline then to the diagnosis of castration or if clinically indicated	–	–
Bone CT	×	Baseline then/3 months	–	–	× bone metastases diagnosis	Baseline then by/4 months from week 16
Thoracic CT	×	“CT Scan”: Baseline then/3 months	× or thoracic radio	Baseline then to the diagnosis of castration or if clinically indicated	× diagnosis measurable metastases according to RECIST 1.1	Baseline then by/4 months from week 16
Abdomen and pelvis CT	×		×			
MRI	–	–	–	–	× diagnosis measurable metastases according to RECIST 1.1	Baseline then by/4 months from week 16
PSA	×	Baseline then/3 weeks in the docetaxel group/3 months in the ADT group	×	Baseline then/3 weeks in the docetaxel group/3 months in the ADT group	×	Baseline then 1/month the first year then 1/2 months

**Table 7:** Assessment exams in former phase III trials in patients who are resistant to castration in the first line setting.

	**TAX 327** **(****73****)**		**COU-AA 302** **(****74****)**		**PREVAIL** **(****75****)**	
	**Type of evaluation**	**Time**	**Type of evaluation**	**Time**	**Type of evaluation**	**Time**
Clinical exam	×	/3 weeks	×	Baseline and pre-specified visits	×	Baseline and pre-specified visits
Bone scan	–	–	×	CT or MRI and bone scan every 8 weeks during the first 24 weeks and then every 12 weeks beyond	×	CT or MRI and bone scinti at the time of screening, at weeks 9, 17, and 25, and every 12 weeks thereafter
Thoracic CT	–	–	×		×	
Abdominal and pelvic CT	–	–	×		×	
MRI	–	–	×		×	
PSA dosage	×	/3 weeks	×	Baseline and pre-specified visits	×	Unspecified time. Discontinuation of treatment on isolated elevation of the PSA not recommended
Unspecified imaging and other	×	All 6–9 weeks repeated after 4 weeks if response	_	_	_	_

**Table 8:** Assessment exams in former phase III trials in patients who are resistant to castration in subsequent lines.

	**ALSYMPCA** **(****76****)**		**TROPIC** **(****77****)**		**COU-AA-301** **(****78****)**	
	**Type of evaluation**	**Time**	**Type of evaluation**	**Time**	**Type of evaluation**	**Time**
Clinical exam	×	Baseline and follow-up	×	Baseline and at each injection	×	–
Bone scan	–	–	–	–	–	–
Thoracic CT	–	–	–	–	×	–
Abdomen and pelvic CT	–	–	–	–		
MRI	–	–	–	–		
PSA dosage	×	After the 12th week	×	–	×	–
Unspecified imaging and other	ALP 1st symptomatic bone events	After the 12th week for ALP	×	–		

#### Response to Treatment

To date, limited data are available on the use of modern imaging in the evaluation of therapeutic response. This is particularly the case with fluciclovine-PET/CT.

##### Choline-PET/CT

Few studies have been published regarding the evaluation of therapeutic response with choline-PET/CT. In a retrospective study of 172 consecutive patients with BCR, choline-PET/CT was positive in 80% of cases, resulting in a management change in 43.6% of cases (79).

Maines et al. evaluated the role of choline-PET/CT in monitoring the response to enzalutamide in 30 patients with metastatic castration-resistant prostate cancer (mCRPC). The authors observed that the maximum standardized uptake value (SUV_max_) measured in choline-PET/CT before treatment with enzalutamide was significantly related to survival without BCR, survival without radiological progression, and overall survival (OS) (80). De Giorgi et al. evaluated choline-PET/CT in the assessment of response to abiraterone in 43 patients. There was a discrepancy between PSA response and choline-PET/CT response in 52% of the patients, and only PET was associated with progression-free survival and OS, in the multivariate analysis (81). Studies were less conclusive in docetaxel response assessment, with disparate findings either in favor of an added value of choline-PET/CT in comparison with PSA kinetics (82), or of limited value in comparison to response evaluation criteria in solid tumors (RECIST) 1.1 criteria (83).

##### PSMA-PET/CT

In a study of 262 patients, 336 PSMA-PET/CTs were performed and detected disease progression and resistance to castration in 100% of cases. A diagnosis of extra-prostatic disease was made at baseline in 53.2% of cases before starting any treatment (84). Albisinni et al. studied the clinical impact of PSMA-PET in patient management and found a change in the treatment plan for 76% of patients (85). This new imaging modality is very promising and might be at the center of treatment planning in the BCR setting in the future, especially for oligometastatic patients.

PSMA-PET has opened new therapeutic avenues. PSMA-positive mCRPC can be treated with specific inhibitors such as Lutetium-177 [177Lu]-PSMA-617. An open-label single-center phase 2 trial from the Peter MacCallum Cancer Center in Melbourne, Australia has shown high response rates, low toxic effects, and a reduction of pain in men with mCRPC who have progressed after conventional treatments (86).

As PSMA-PET/CT is increasingly adopted in clinical trials and routine practice worldwide, a unified language for image reporting is urgently needed.

##### NaF-PET/CT

In the US registry study on PET, in 3,531 patients with PCa, NaF-PET/CT impact management replaced the use of other advanced imaging techniques in 50% of cases (87). Treatment plan modification was recommended in 76% of cases (87). Furthermore, Etchebehere et al.'s (88) recent study in 42 patients with castration-resistant PCa during radium-223 treatment showed that NaF can be a predictive marker for OS and occurrence of bone-related events.

##### Bone-scan

Concerning the monitoring of patients with bone metastases, evaluation of response to treatment with a bone scan is also challenging because of the flare-up effect that can occur up to 12 weeks after the beginning of treatment. This is why intensity changes or minor changes in the extent of existing lesions are non-specific, and should not be considered as a determinant of progression (37). Bone scintigraphy with or without SPECT-CT only distinguishes “progression/non-progression” or “new lesion/absence of new lesions” without the possibility of early identification of “response/non-response.” For exclusive bone metastases, bone progression is defined by the PCa Clinical Trials Working Group 3 (PCWG3) (89) as the occurrence of at least two new lesions. The PCWG3 emphasized that only positivity on the bone scan defines metastatic disease to the bone.

##### MRI

Regarding bone MRI, there have been few studies with specific evaluation of the response in bone metastatic PCa patients (90, 91). Reischauer et al. found that the mean diffusivity of the lesions increased considerably after hormone therapy. There was also a spatial heterogeneity in the metastases, as the diffusivity of water increases at the center of the lesions. An important challenge is attempting to evaluate the activity of the disease through the MRI sequences. Some abnormalities, such as false pseudo-progression in the T1-W sequence, may exist due to an edematous reaction of the bone marrow related to tumor destruction and inflammation (92). In MRI, T1 and saturated fat sequences improve the identification of the radiological response due to the recognition of the return of the fat from the responder marrow. In patients with castration-resistant PCa, T1-W MRI more than doubled the proportion of patients with measurable lesions (29% on the scanner vs. 66% on the MRI) and allowed discrimination of the radiological responses between complete response, partial response, stability, or progression. Thus, MRI size/volume measurements are useful for evaluating the response beyond bone scintigraphy, which identifies progression only (93). The results of diffusion MRI are heterogeneous. A confounding factor in the interpretation of the results is the concomitant use of inhibitors of bone resorption, such as bisphosphonates or anti-RANKL. Their individual effects have not yet been described in the interpretation of bone parameters in functional MRI.

WB-MRI is more reliable in identifying and measuring bone disease than CT (94) or scintigraphy (95). Morphological approaches to MRI using diffusion and contrast have recently been extended to bone and significantly contributed to improving the diagnostic performance of WB-MRI (96). MRI and PET-CT allow quantification of disease and categorization of patients into “complete response,” “partial response,” “stable disease,” or “progressive disease” and therefore provide objective evidence of therapeutic benefits. In the future, hybrid PET/MRI scanners may play a key role in the imaging of metastatic bone disease.

## Discussion

### Summary of the Main Findings

The most thoroughly validated tests in clinical trials and in clinical practice are the “good old tests” such as bone scan and contrast-enhanced thorax, abdomen, and pelvis CT.

Nevertheless, most of the evidence based for bone scan and CT are old and retrospective. There is actually a much larger amount of prospective data for “modern” PET/CT. Prospective data also shows clear superiority of PSMA-PET or fluciclovine-PET to choline-PET. Data from a new study suggests that PSMA-PET imaging is superior to fluciclovine-PET for detecting biochemical recurrence in men with prostate cancer (44). However, whether one of these radiotracers improves patient survival over the other is unknown and further research is needed to determine which has the greater effect.

The new tracers do not have the same accessibility and need further validation in clinical trials to evaluate their benefits and clarify their usage in routine clinical practice. It is important that we consider the imaging requirements of the PCWG as this defines the imaging definitions for subjects in those clinical trials. EORTC algorithms also need to be considered for the imaging strategies. Experts in nuclear medicine and radiology should be represented in medical decision-making teams for PCa.

### Brief Idea for the Future

**Figure 3:**
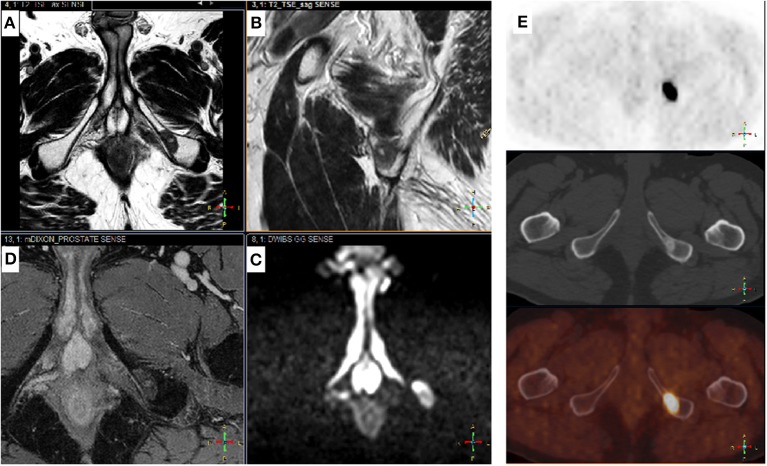
Complementary Choline-TEP and pelvic MRI. Patient with a history of Gleason 7 (4 + 3) prostatic adenocarcinoma treated by radical prostatectomy 9 years ago (pT3aN0M0R1). PSA recurrence 5 years later followed by EBRT, external beam radiation therapy. Three years later, rising PSA from 0.5 to 1.4 ng/mL in 6 months. Prostate MRI performed before 18-F choline-PET-CT shows no sign of recurrence in the prostatectomy bed, but detected a 17-mm suspicious bone lesion in the left ischiopubic branch. Typical signal on axial **(A)** and sagittal **(B)** T2-w images were low and homogeneous; high on b-1000 axial diffusion-weighted images (DWI) **(C)** and high on late T1-w dynamic contrast-enhanced (DCE) images **(D)**, corresponding to high cellular density and hypervascularization, respectively. 18F choline-PET-CT **(E)**: axial slices of the pelvis showing an osteosclerotic lesion on the right ischiopubic branch with high uptake of the tracer. From top to bottom: PET, CT, PET-CT.

Modern PET/CT have a high overall sensitivity, whereas WB-MRI has a high specificity. They are therefore complementary techniques, hence the interest in PET/MRI (97) (Figure 3). The future is certainly in the combination of imaging techniques, and a recent EORTC consensus proposed clinical trials that use modern imaging methods to evaluate the benefits of metastasis-directed therapies. The EORTC imaging group suggested a clinical algorithm to integrate modern imaging methods into care pathways to identify oligometastatic disease at the various PCa stages (6). Recently, Eiber et al. proposed a molecular imaging tumor, node, and metastasis system (miTNM Version 1.0) as a standardized reporting framework for PSMA-ligand PET/CT or PET/MRI (98). Padhani et al. proposed the METastasis Reporting and Data System for PCa, which provides imaging recommendations designed to promote standardization and reduce variations in MRI acquisition, interpretation, and reporting in advanced PCa, not only at the beginning of treatment, but also as the disease progresses. However, this technique requires validation in clinical trials (99).

### Developing Landscape for Non-metastatic Castration-Resistant PCa

Non-metastatic CRPC (nmCRPC) prevalence has been estimated at 7% of PCa in the European Union (100). Owing to the advent of modern imaging, the prevalence of this subgroup has declined. These newer imaging agents i.e., fluciclovine or PSMA can identify local recurrence or metastases at PSA levels far below the traditional PSA threshold for other imaging modalities such as CT or bone scan. Some of the new PET agents can identify metastatic lesions at PSA values as low as 0.2–0.5 ng/mL, allowing the prompt identification of M1 CPRC (100). This is interesting given the recent randomized studies in this field (SPARTAN and PROSPER), which show the efficacity of apalutamide (101) and enzalutamide (102). The metastasis-free survival of patients with nmCRPC was previously estimated as ~25–30 months, but can now be significantly prolonged using combination next-generation hormone therapy with ADT (100). The use of PSA doubling time to determine risk for progression can guide the appropriate timing for starting therapy. For instance, a PSA doubling time ≤ 10 months is appropriate to initiate therapy. There is no currently established standard of care option for treatment of this population, hence the need for enrollment in clinical trials (103).

## Conclusion

This is a wide critical literature review of the imaging methods in prostatic cancer, focused especially on metastasis detection and treatment response assessment. In this common but paradoxical cancer, many imaging methods are available, but the recommendations are not clearly established.

**Figure 4:**
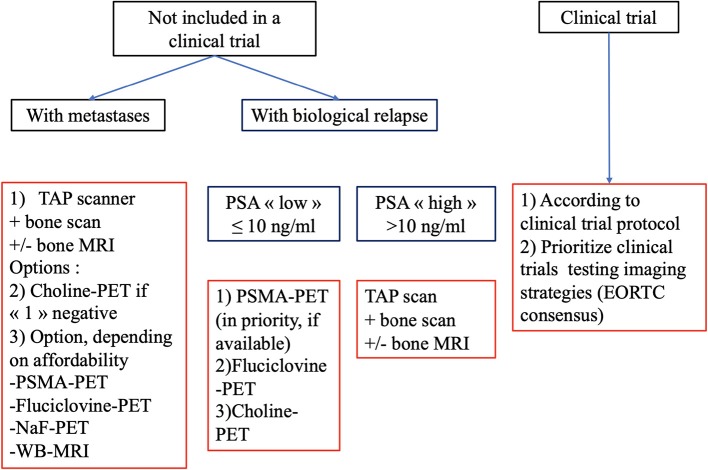
Flow chart of recommended imaging in evaluating metastatic PCa in routine practice.

Modern PET/CT imaging techniques provide better sensitivity and specificity of metastasis detection, especially in cases of biochemical recurrence with low values of prostate specific antigen. Conventional imaging i.e., bone scan, CT-scan or MRI still have a role, especially in localized and metastatic disease for the follow-up of patients under systemic treatment. We have summarized our findings in Figure 4 with a proposed algorithm to facilitate communication between imagers and clinicians, in order to select the most-validated imaging. Validated clinical trials with new radiotracers are needed.

## Author Contributions

AT, EG, and NP did the concept of the review. AT and EG acquired the data and select the articles of interest in the literature. NP controlled the quality of data. AT, EG, CB, and PP prepared the manuscript. PP and CB selected images of interest and did the figures. AV, JO, and DP reviewed the manuscript.

### Conflict of Interest

The authors declare that the research was conducted in the absence of any commercial or financial relationships that could be construed as a potential conflict of interest.

## Footnotes

^1^https://www.nccn.org/professionals/physician_gls/pdf/prostate.pdf. Version 4.2019, 08/19/19 © 2019 National Comprehensive Cancer Network® (NCCN®), All rights reserved.
